# Development of a Biocontrol Method Applying Bacteriophage-Containing Aerosol against *Mycobacterium tuberculosis* Using the Bacteriophage BTCU-1 and *M. smegmatis* as Models

**DOI:** 10.3390/microorganisms7080237

**Published:** 2019-08-03

**Authors:** Chun-Chieh Tseng, Dan Chi Chang, Kai-Chih Chang

**Affiliations:** 1 Department and Graduate Institute of Public Health, Tzu Chi University, Hualien 97071, Taiwan; 2 Department of Laboratory Medicine and Biotechnology, Tzu Chi University, Hualien 97071, Taiwan

**Keywords:** tuberculosis, *Mycobacterium smegmatis*, bacteriophage, bioaerosol, biocontrol, air sampling

## Abstract

The application of bacteriophages for biocontrol has attracted increasing attention. Here, we applied ϕBTCU-1 as a model phage to develop a method for controlling *Mycobacterium tuberculosis* (MTB) by using a bacteriophage-containing aerosol in a chamber study. The soil-isolated ϕBTCU-1 can infect both MTB and *Mycobacterium smegmatis*. Our study used *M. smegmatis* as an MTB surrogate for safety reasons. Among all the evaluated air samplers, the Andersen impactor was chosen to evaluate the bactericidal efficiency of ϕBTCU-1 against *M. smegmatis* since the recovery rates of the Andersen impactor were 1.5 to 10.6 times higher than those of sampling filters. When airborne ϕBTCU-1 with the highest concentration of 10^9^ PFU/m^3^ challenged *M. smegmatis* (10^5^ CFU/m^3^) for 10 s, no *M. smegmatis* colony was recovered from the culture medium. For surface decontamination, no colony of *M. smegmatis*, which started at 1000 CFU/plate (63.6 cm^2^), was recovered when exposed to higher ϕBTCU-1 concentrations (>10^9^ PFU/m^3^) for 60 min. Bacteriophages may be useful for reducing MTB contamination in the air or on hard surfaces. The method we have established suggests that the biocontrol method may be an alternative approach or may be combined with other disinfection methods to prevent MTB infection.

## 1. Introduction

Tuberculosis (TB) is an infectious disease caused by *Mycobacterium tuberculosis* (MTB). In Taiwan, the TB incidence (43.9/100,000) was 15 times higher than that in the United States (2.9/100,000) in 2016 [1,2]. Although the incidence rate and mortality rate both decreased by approximately 40% in Taiwan, the number of new cases (10,328) in Taiwan in 2016 was still higher than that in the US (9287) [1,2].

TB is mostly transmitted by contact with infected persons who release airborne MTB through coughing, sneezing, or spitting. When TB patients visit the hospital, a varied concentration range of MTB, from 1.9 to 5.2 × 10^4^ copy/m^3^, can be detected by a real-time quantitative polymerase chain reaction during air sampling periods [3]. These concentrations do not represent the detected MTB that were viable because of the limitations of polymerase chain reaction. The detected MTB concentrations still pose a potential hazard to healthcare workers, especially as the infectious dose (ID_50_) of MTB is very low, estimated at <10 bacilli in humans [4]. Therefore, the control of MTB in a hospital environment is still an important issue worldwide.

To control TB transmission, ultraviolet germicidal irradiation (UVGI) is the most commonly used method for the inactivation of airborne MTB [5,6]. Basically, a satisfactory inactivation efficiency of MTB can be achieved when a sufficient UV dose (UV intensity multiplied by irradiation time) is applied. However, UVGI cannot completely penetrate shadowed crevices; it can also have adverse effects on humans. Consequently, alternative strategies for preventing the spread of MTB are needed.

Bacteriophage (phage) therapy has been used since 1919, but was almost abandoned after broad-spectrum antibiotics appeared [7]. Phages are natural parasites of bacteria and are highly host specific. Since MTB can be antibiotic resistant and, thus, can cause serious health concerns, the successful use of phages for TB treatment in animal models suggests that this strategy has the potential to be applied to humans [8,9,10]. In addition to clinical treatment, phages also have the potential for different applications. For example, phages have been used to treat foods and as disinfectant agents [11,12,13]. Our study group also successfully generated phage aerosols in intensive care units (ICUs) against nosocomial transmission caused by carbapenem-resistant *Acinetobacter baumannii* (CRAB) [14]. Although we observed that the number of new CRAB cases in ICUs decreased [14], the aerosol generation conditions, including the aerosolized phage concentration and aerosol generation time, have not been optimized. The decontamination efficiency should be improved if these conditions are to be optimized.

To date, the application of a phage as an aerosol disinfectant against airborne MTB has never been reported. Consequently, the purposes of this study were to develop a method using a model phage to determine the phage concentration and aerosol generation time when aerosol decontamination was conducted. The model phage in this study was ϕBTCU-1, a mycobacteriophage isolated from the soil. ϕBTCU-1 belongs to the *Siphoviridae* family, and its genome has been fully sequenced [15]. The lytic activity of ϕBTCU-1 on clinical MTB strains was also investigated in our previous study [15]. This phage has a broad host range, including seven clinical MTB isolates and their surrogate organism, *Mycobacterium smegmatis* [15]. These advantages make ϕBTCU-1 a good model phage to demonstrate the potential efficiency of decontaminating MTB in the environment. For safety reasons, we used *M. smegmatis* as the surrogate of MTB since MTB has high virulence and requires a long generation time. Next, the inactivation effects of ϕBTCU-1 phage aerosols were studied from two perspectives. One aimed to investigate which bioaerosol sampler is most suitable for collecting airborne *M. smegmatis* to conduct a subsequent bactericidal experiment by phage aerosol. The other aimed to evaluate the bactericidal efficiency when ϕBTCU-1 phage aerosol was applied against the survival of *M. smegmatis*, including when these bacteria were in the air or on the agar surface.

## 2. Materials and Methods

### 2.1. Phase One: Phage Aerosol against Airborne M. smegmatis

#### 2.1.1. Bacterial Host Strain and Culture

ϕBTCU-1 can infect both MTB and *M. smegmatis*. A nonpathogenic and fast-replicating *M. smegmatis* MC^2^ (ATCC 70084) was used as a bacterial host, instead of the pathogenic strain, to simulate the bactericidal effect of ϕBTCU-1 on MTB. To generate aerosols, *M. smegmatis* was inoculated in Middlebrook 7H9 medium (Difco, Detroit, MI, USA) and then incubated for 48 h at 37 °C. When *M. smegmatis* was grown to stationary phase for 48 h, microbe pellets were collected, aseptically washed with phosphate-buffered saline (PBS), and transferred to a centrifuge tube, which was capped and centrifuged (800× *g*, 5 min). Subsequently, the supernatant was discarded and the pellets were resuspended in sterile distilled water for the preparation of bacterial aerosol spray suspensions.

#### 2.1.2. Phage Preparation

ϕBTCU-1 phage was isolated from a soil sample from Tzu Chi University, Hualien, Taiwan. A high-titer stock of ϕBTCU-1 phage with 10^10^ plaque-forming units (PFU)/mL was prepared via plate lysis and elution. The phage purification process involved centrifugation at 6000× *g* for 10 min to remove the remaining bacterial debris. In addition, the phage supernatant was passed through a 0.22 μm filter (Millipore Corporation, Bedford, MA, USA) to yield a phage solution free of host cells. Further purification processes are not considered in this study because we need high titer phage stocks and many techniques may affect phage recovery [16]. To maintain phage infectivity, a phage buffer containing 10 mM Tris-HCl, 10 mM MgSO_4_, 68.5 mM NaCl, and 1 mM CaCl_2_ was prepared. The double-agar-layer method [17] used the same phage buffer for plaque observation and enumeration in triplicate. All plates utilized for the double-agar-layer method were incubated at 37 °C for 24 h.

#### 2.1.3. Effect of Temperature on ϕBTCU-1 Stability

To determine which temperature is appropriate to store the phage stock solution, the ϕBTCU-1 stock (10^9^ PFU/mL) was inoculated in the phage buffer, divided into 1 ml vials and stored at −20 °C or 4 °C. Each solution vial was stored at two different temperatures with 0.6% glycerol and inoculated for plaque assay at various time points for up to 60 days. The vials taken from −20 °C storage were thawed at 4 °C until the samples melted into a liquid and then were removed at room temperature. All vial samples taken from the refrigerator were immediately used in the double-agar-layer method within 15 min. In addition, the vials used in this test were all discarded and never refrozen. In addition, considering that ϕBTCU-1 is used to decontaminate MTB at room temperature, we also evaluated ϕBTCU-1 stability at room temperature (25–27 °C). When phage aerosol sprays in the environment, we do not expect that this phage can decontaminate MTB for a long-term period. Consequently, we only evaluated ϕBTCU-1 stability at room temperature for one week. We used the Ct/C_0_ ratio to determine the ϕBTCU-1 stability at different temperatures, where Ct and C_0_ are the phage culturable concentrations recovered from the solution that had been stored for t day and 0 day, respectively.

#### 2.1.4. *M. smegmatis* Aerosol Preparation and Test System

**Figure 1:**
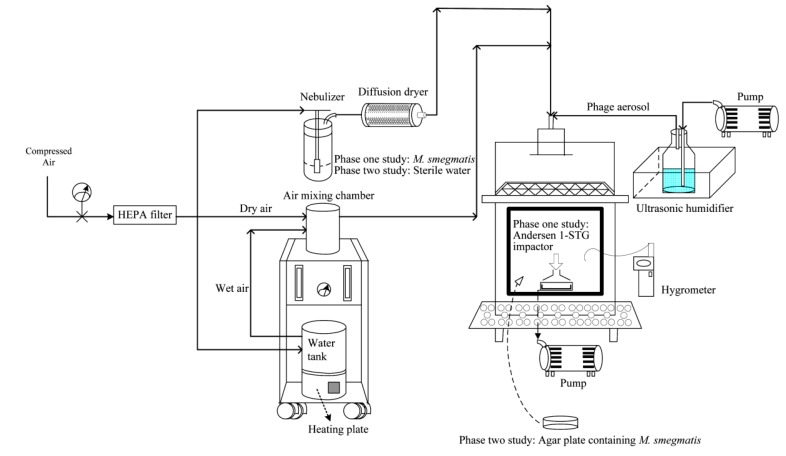
Schematic diagram of the bioaerosol test system for (a) collection efficiency of tested bioaerosol samplers for collecting airborne *M. smegmatis*, (b) reduction of airborne *M. smegmatis* by ϕBTCU-1 aerosols (phase one study), and (c) the bactericidal effect of ϕBTCU-1 aerosols on *M. smegmatis* colonies on an agar surface (phase two study). The suspension in the nebulizer was *M. smegmatis* and the sampler in the test chamber was an Andersen 1-STG impactor in the phase one study. In the phase two study, sterile water was used to replace *M. smegmatis* in the nebulizer and an agar plate contaminated with *M. smegmatis* was used to replace the Andersen 1-STG impactor in the chamber.

The aerosol generation system was similar to that of our previous study [18]. Airborne *M. smegmatis* was generated by a Collison three-jet nebulizer (BGI Collison Nebulizer, BGI Inc., Waltham, MA, USA), at 3 L/min into the test chamber, with a volume of 97 L (Figure 1). The concentration of *M. smegmatis* in the nebulizer was stable for up to 60 min of aerosol generation (Figure S1). The *M. smegmatis* aerosol was combined with an entrance air stream (flow rate = 50 L/min) toward the test chamber inlet. To simulate a moderate relative humidity (RH) of 55% in the test chamber, the water vapor content in the gas stream was adjusted by changing the flow rate ratio of the humidified and dry gas stream. A hygrometer (Rotronic AG, Bassersdorf, Switzerland) was used to measure the actual RH in the test chamber.

#### 2.1.5. Test Sampler and Sample Processing

To investigate which bioaerosol sampler was appropriate to collect airborne *M. smegmatis*, the bacterial colony survival rate after sampling on agar media was evaluated by an Andersen 1-STG impactor (Andersen Samplers, Inc., Atlanta, GA, USA), a BioSampler (SKC Inc., Connellsville, PA, USA), a gelatin filter (Sartorius AG, Gottingen, Germany), and a Nuclepore filter (Nuclepore®, Costar Corp., Cambridge, MA, USA). In this test, the concentrations of airborne *M. smegmatis* generated in the chamber were maintained at 10^2^–10^5^ CFU/m^3^. The sampling flow rates of the Andersen impactor and BioSampler were 28.3 and 12.5 L/min, respectively. The BioSampler was filled with 20 ml of deionized water to collect airborne *M. smegmatis.* For the gelatin filter and Nuclepore filter sampling, the sampling flow rate was 20 L/min. A high flowrate was chosen because these filters are commonly used to detect MTB and viruses at low concentrations [19,20]. Both filter samplers were supported by cellulose pads and consisted of a 3.0 μm and 0.4 μm pore size for the gelatin filter and Nuclepore filter, respectively. As we generated different bacterial aerosol concentrations in the chamber, the sampling time of these three samplers was adjusted (from 5 s to 60 min) according to the different levels of aerosol concentration.

After sampling, the plates of Middlebrook 7H11 agar from the Andersen 1-STG impactor were incubated at 37 °C for 48 h. For BioSampler sampling, the remaining volumes of deionized water inside the sampler were measured. After gelatin filter sampling, the filter was dissolved on the 7H11 agar surface for further incubation. For the Nuclepore filter, the filters were eluted by rinsing with 1 ml of sterile deionized water and then gently vortexed by a rotator (Vortex-2 Genie, Scientific Industries Inc., NY, USA) for 30 s. Subsequently, the deionized water from the BioSampler and the filter eluate were both inoculated on 7H11 agar at 37 °C for 48 h. Finally, airborne bacterial counts in three bioaerosol samplers were calculated based on the dilution ratio, plated volume, sampling time, and sampling flow rate.

#### 2.1.6. Calculation of the CR Value for Air Sampling of *M. smegmatis*

Our previous study applied culturability recovery (CR) as a bacterial recovery indictor [18] to investigate which bioaerosol sampler may cause less damage to the microbe’s culturability. The advantage of CR is that we can adjust the initial culturability to determine the biological efficiency of the bioaerosol sampler. The CR values are defined as follows:

(1) $$\mathrm{CR}=\frac{C_{\text{sampler}}}{C_{\text{nebulizer}}}$$

where C_sampler_ is the CFU/m^3^ determined for 7H11 agar (i.e., the number of culturable *M. smegmatis* per cubic meter of air that passed through the sampler), and C_nebulizer_ is the CFU/ml in the nebulizer (Figure 1) determined for 7H11 agar (i.e., the amount of culturable *M. smegmatis* in the nebulizer).

#### 2.1.7. Generation of Phage Aerosols against Airborne *M. smegmatis*

In the phase one study, the phage aerosol was generated for the inactivation of airborne *M. smegmatis*. The ϕBTCU-1 aerosol was generated by an ultrasonic humidifier (Lab Mister, DDON LTD Inc., Taiwan) containing the phage buffer (10 mM Tris-HCl, 10 mM MgSO_4_, 68.5 mM NaCl, and 1 mM CaCl_2_). The ultrasonic humidifier can generate ultrasonic vibrations that break water molecules to create water droplets. With respect to phage stability, we kept the vibrating water at a low temperature and the concentration of phage solution in the ultrasonic humidifier was determined by plaque assay at each generation time point. The phage concentration in our ultrasonic humidifier was stable for up to 60 min of aerosol generation (Figure S2).

The humidifier applied a metal diaphragm to generate an ultrasonic frequency to vibrate and then create phage droplets in a cool fog form at 5 L/min (Figure 1). The concentrations of phage stock solution in the humidifier were 1 × 10^7^, 1 × 10^8^, and 1 × 10^9^ PFU/mL, which corresponded to airborne concentrations of 1 × 10^7^, 1 × 10^8^, and 1 × 10^9^ PFU/m^3^, respectively (Figure S3). These airborne phage concentrations were determined by the BioSampler filled with 20 mL of the phage buffer for sampling for 10 min. To challenge phage aerosols, three different bacterial concentrations were investigated at RH 55%. The *M. smegmatis* aerosols in the chamber were 1 × 10^3^, 1 × 10^4^, and 1 × 10^5^ CFU/m^3^, respectively. Such high concentrations of MTB are very rare in clinical settings [3]. To obtain countable colonies from agar plates, airborne samples of each bacterial aerosol were taken by an Andersen 1-STG impactor from 5 s to 30 s in sequence, first without and then with phage aerosol. The survival rates of *M. smegmatis* were determined by colony counts when challenged with phage aerosol (N_t_) against colony counts when challenged with water fog without phage (N_0_). Finally, the log reduction in colony counts was calculated as the log_10_ of N_t_/N_0_.

### 2.2. Phase Two: Phage Aerosol against M. smegmatis Colonies on an Agar Surface

In a phase two study, we investigated whether phage aerosols can be applied to inactivate *M. smegmatis* on an agar surface. Based on our aerosol test system (Figure 1), the airflow generated from the humidifier toward the test chamber inlet permitted the phage aerosols to settle directly onto the agar plate spread with *M. smegmatis.* The phage droplets created in a cool fog (5 L/min) were combined with an entrance air stream (flow rate = 6 L/min) toward the test chamber inlet. Consequently, the total flowrate of 11 L/min in the phase two study can result in 6.8 air changes per hour (ACH) to simulate the recommended ACH in a hospital ward [21]. In the phase two study, the phage and *M. smegmatis* culturable methods were the same as in the phase one study. *M. smegmatis* at stationary phase after 48 h of incubation was also obtained to conduct our phase two study. However, the phage and *M. smegmatis* concentrations evaluated in the phase two study were adjusted for surface evaluation. Before applying phage aerosols to inactivate the bacterial colonies on the agar surface, we also evaluated the deposition of phage aerosols on a growing lawn of the bacterial host. First, the concentration of *M. smegmatis* was determined by determining the optical density at 600 nm (OD_600_ = 0.6). Subsequently, the double-agar-layer method was applied for this test. However, the top layer of agar made with the 7H9 medium only contained the bacterial host, not the phage. Finally, the plates containing host lawns were exposed to phage aerosols for 10, 30, and 60 min.

After the phage deposition test, the concentrations of phage aerosols were 1 × 10^8^, 1 × 10^9^, and 1 × 10^10^ PFU/m^3^ against *M. smegmatis* with 10, 100, and 1000 CFU on each agar plate (63.6 cm^2^), respectively. In fact, 1000 CFU per agar plate is the highest concentration we can observe and clearly count on the medium. Bacterial concentrations higher than 1000 CFU per agar plate would be overloading and difficult to count. Therefore, the agar plates spread with *M. smegmatis* were directly exposed to the phage aerosol or only the water fog for 10, 30, and 60 min, respectively. After aerosol exposure, the individual agar plates with bacterial colonies were directly incubated for 48 h at 37 °C. Finally, the culturable bacterial counts from the agar plate were calculated based on the plated volume and dilution ratio. The survival rate was calculated as the log_10_ of N_t_/N_0_, where N_0_ is the number of bacterial colonies recovered from the water fog challenge and N_t_ is the number of colonies from the phage aerosol challenge.

### 2.3. Statistical Analysis

The differences in the phage concentrations between the two storage temperatures were determined using the Mann–Whitney–Wilcoxon test. The CR values of different bioaerosol samplers for collecting different concentrations of *M. smegmatis* were compared using the Kruskal–Wallis test, followed by Dunn’s multiple comparison test to detect statistically significant differences (*p* < 0.05). The same statistical method was also used to compare (1) the differences in the Nt/N_0_ values among three phage concentrations, (2) the differences in the Nt/N_0_ values among three *M. smegmatis* concentrations, and (3) the differences in the Nt/N_0_ values among three sampling times or exposure times.

## 3. Results

### 3.1. Effect of Temperature on ϕBTCU-1 Stability

**Figure 2:**
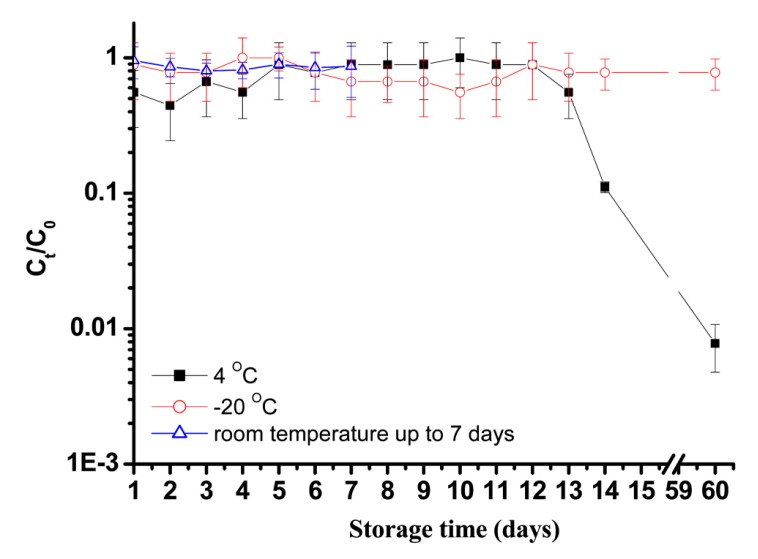
Stability of ϕBTCU-1 stored in phage buffer at −20 °C and 4 °C. ϕBTCU-1 stability at room temperature (25–27 °C) was also evaluated, although it was only stored for 7 days. The vials used in this test were all discarded and never refroze again. These experiments were repeated three times and the data shown are the mean ± standard error of the mean.

Before phage aerosol decontamination, these phage “disinfectants” are often not used immediately. To determine which storage temperature is appropriate, the ϕBTCU-1 stock (10^9^ PFU/ml) was inoculated in the phage buffer. Figure 2 shows the stability of ϕBTCU-1 stored in phage buffer at −20 °C and 4 °C for up to 60 days. When the phages were stored in phage buffer at −20°C and 4 °C for 14 days, they retained 100% and 20% infectivity, respectively. When the storage time was extended to 60 days, the phages still retained 100% at −20°C, but only 1.4% of phages were retained at 4 °C. If the storage time was less than 14 days, there was no significant difference in phage concentrations between −20 °C and 4 °C storage. ϕBTCU-1 was much more stable when the sample was frozen. If the sample was only refrigerated, ϕBTCU-1 was unstable for more than 2 weeks of storage. In addition, ϕBTCU-1stored at room temperature was quite stable for 7 days.

### 3.2. Collection Efficiency of Tested Bioaerosol Samplers

**Figure 3:**
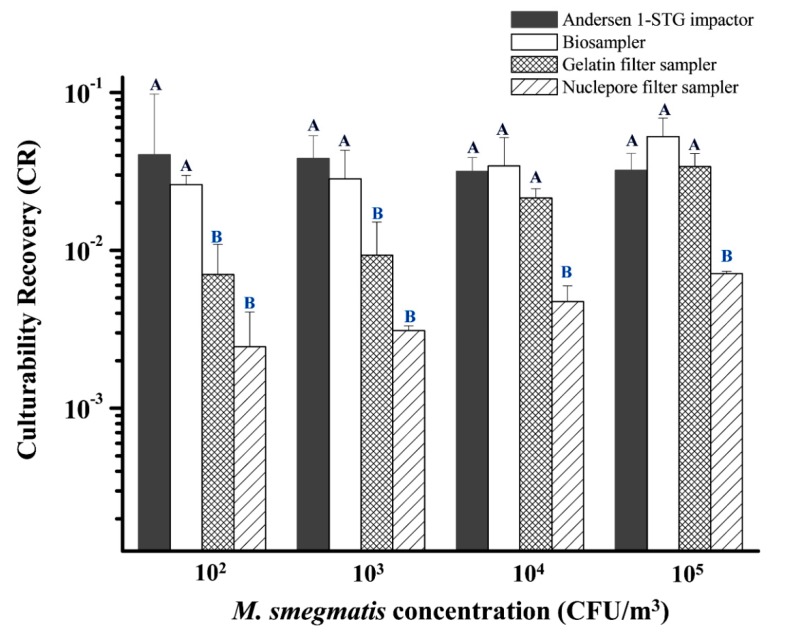
Relative culturable collection efficiencies of bioaerosol samplers for capturing airborne *M. smegmatis*. The indicator CR was used to adjust the initial culturable concentration in the nebulizer to determine the biological performance of the tested samplers. Samplers with higher CR values can preserve more culturability of *M. smegmatis* under the sampling conditions. Samplers with the same letter (e.g., A versus A; B versus B) have similar CR values that are not significantly different (*p* > 0.05, Dunn’s multiple comparison test). However, if samplers with different letter (e.g., A versus B), their CR values are significantly different. Experiments were performed in triplicate and the data shown represent the mean ± standard error of the mean.

Figure 3 shows the collection efficiency of four different bioaerosol samplers for collecting varied concentrations of airborne *M. smegmatis*. A combination of all bioaerosol samplers showed that the CR value of 10^5^ CFU/m^3^ was significantly higher than that of 10^2^ CFU/m^3^ (*p* = 0.023). The CR value of the Nuclepore filter was always the lowest relative to the other three bioaerosol samplers. Another gelatin filter performed as well as the Andersen 1-STG impactor and BioSampler when higher concentrations of *M. smegmatis* (10^4^ and 10^5^ CFU/m^3^) were evaluated. However, if lower *M. smegmatis* concentrations (10^2^ and 10^3^ CFU/m^3^) were applied, the gelatin filter had significantly lower CR values than those of the Andersen 1-STG impactor and BioSampler.

### 3.3. Reduction of Airborne M. smegmatis by ϕBTCU-1 Aerosols

**Figure 4:**
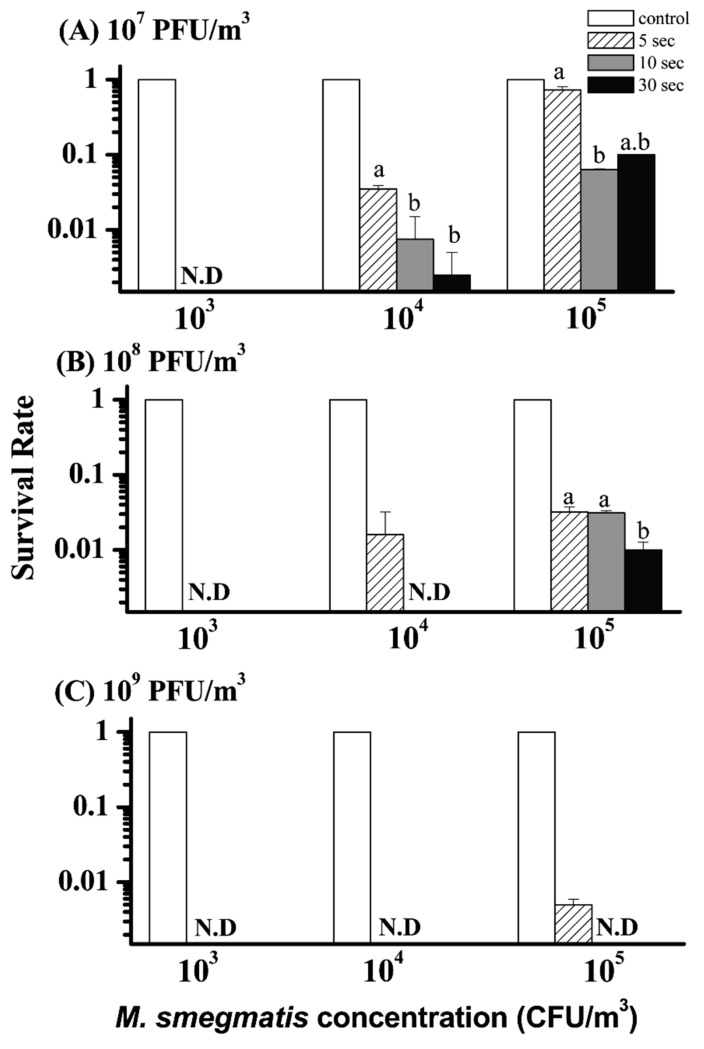
Bactericidal effect of different concentrations, (**A**) 10^7^, (**B**) 10^8^, and (**C**) 10^9^ PFU/m^3^, of ϕBTCU-1 aerosols on different concentrations of airborne *M. smegmatis* in the test chamber at three times of 5, 10, and 30 s sampling by an Andersen 1-STG impactor (phase one study). The survival rate was calculated as the log_10_ of N_t_/N_0_, where N_0_ is the number of bacterial colonies recovered from challenging with water fog and N_t_ is the number of colonies recovered from challenging with ϕBTCU-1 aerosol. The histogram bars with the same letter represent that they have similar survival rates that are not significantly different (*p* > 0.05, Dunn’s multiple comparison test). Experiments were performed in triplicate and the data shown represent the mean ± standard error of the mean. N.D. means not detected.

Next, we investigated the ability of ϕBTCU-1 to reduce the concentration of airborne *M. smegmatis* over different sampling times (Figure 4). In this test, the Andersen 1-STG impactor was used to collect airborne *M. smegmatis* because of its higher CR value and convenience. The survival rates were all 100% for the control groups. In general, ϕBTCU-1 had a good bactericidal effect (>90% inactivation rate) in all test groups except the 5 s sampling time in the low-dose group (10^7^ PFU/m^3^) against 10^5^ CFU/m^3^ of *M. smegmatis* (Figure 4). The lowest concentration of ϕBTCU-1 (10^7^ PFU/m^3^; Figure 4A) exhibited the weakest bactericidal capability. The bacterial survival rate increased as the airborne phage concentration decreased. Application of 10^8^ or 10^9^ PFU/m^3^ of ϕBTCU-1 reduced the concentration of *M. smegmatis* by at least 1 log at all bacterial test concentrations after sampling for 5 s (Figure 4B,C). The effect was even obvious after 30 s sampling, with at least a 2 log reduction in *M. smegmatis* survival rates. Furthermore, the bactericidal efficiency caused by phage infection decreased as the initial bacterial concentration increased. In addition to the phage and bacterial host concentrations, the sampling time was also important for determining the bactericidal effect. Overall, increasing the sampling time decreased the bacterial survival rate. However, the bactericidal effect at different sampling times was not significantly different for the highest phage group (10^9^ PFU/m^3^) because almost all bacterial colonies could not easily recover from all sampling time sets.

### 3.4. Bactericidal Effect of ϕBTCU-1 Aerosols on M. smegmatis Colonies on an Agar Surface

**Figure 5:**
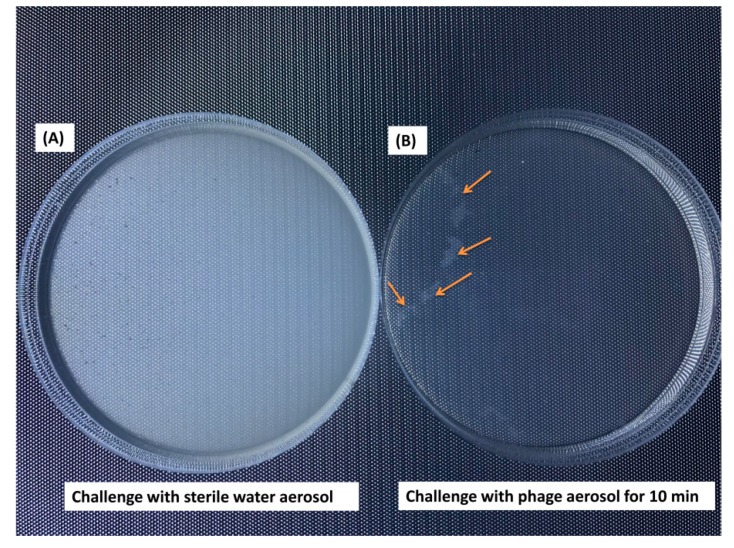
Phage aerosols deposited on plate surfaces with growing lawns of the bacterial host (OD_600_ = 0.6). (**A**) Host bacterial lawn without phage aerosol challenge (sterile water aerosol) and (**B**) host bacterial lawn exposed to phage aerosol (10^8^ PFU/m^3^) for 10 min. Arrows indicate a few areas of host lawn that were not covered by the phage aerosol and still maintained a translucent appearance. Host bacterial lawns exposed to phage aerosol for longer than 10 min demonstrated complete clearance.

Figure 5 demonstrates the deposition of phage aerosols on a growing lawn of the bacterial host. As very similar results were obtained when higher phage concentrations were used, we only demonstrated plate images when the host lawn plate was exposed to phage aerosols at 1 × 10^8^ PFU/m^3^ for 10 min. When the bacterial host lawn was not exposed to phage aerosols, the host bacteria were evenly distributed on the 7H9 medium and showed a translucent appearance (Figure 5A). When the bacterial host lawn was exposed to phage aerosols at 1 × 10^8^ PFU/m^3^ for 10 min, the medium was almost clear, and only a few areas maintained translucence (Figure 5B). This result demonstrated that the time required for deposited phage aerosols to cover all areas of the plates was longer than 10 min. When the exposure time was extended longer than 10 min, the plates demonstrated complete clearance.

**Figure 6:**
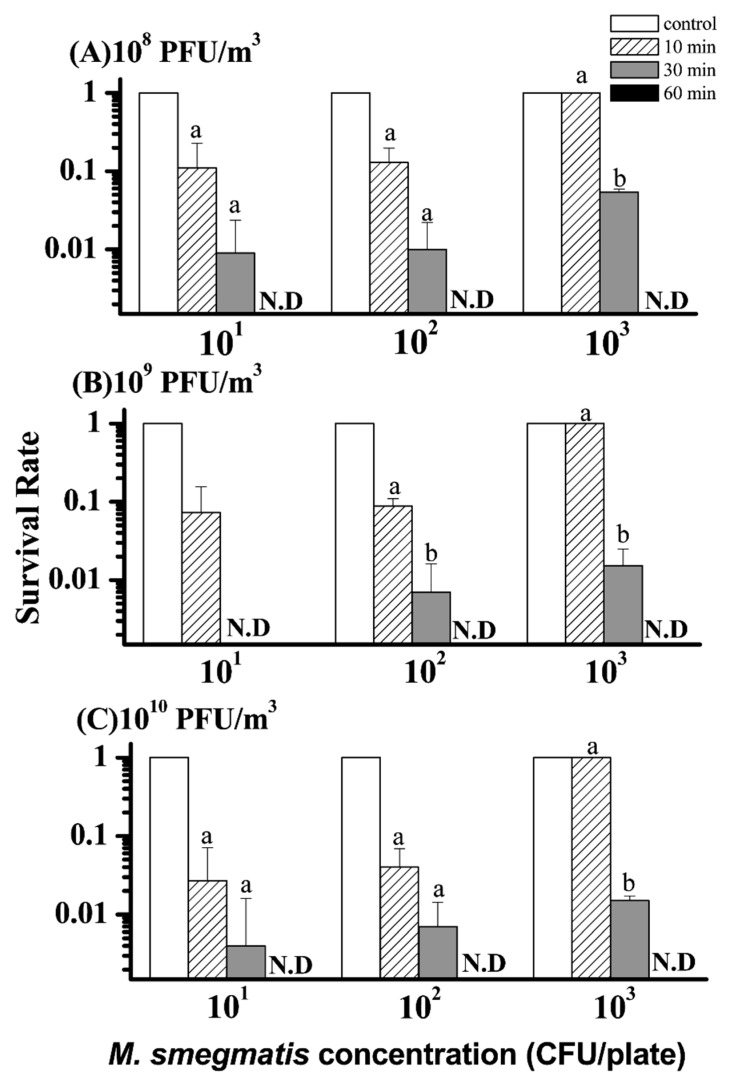
Bactericidal effect of different concentrations, (**A**) 10^8^, (**B**) 10^9^, and (**C**) 10^10^ PFU/m^3^, of ϕBTCU-1 aerosols on an agar plate contaminated with different concentrations of *M. smegmatis* at three exposure times of 10, 30, and 60 min (phase two study). The survival rate was calculated as the log_10_ of N_t_/N_0_, where N_0_ is the number of bacterial colonies recovered from challenging with water fog and N_t_ is the number of colonies recovered from challenging with ϕBTCU-1 aerosols. The histogram bars with the same letter represent they have similar survival rates that are not significantly different (*p* > 0.05, Dunn’s multiple comparison test). Experiments were performed in triplicate and the data shown represent the mean ± standard error of the mean. N.D. means not detected.

The exposure of ϕBTCU-1 aerosol to an agar plate surface contaminated with *M. smegmatis* had a significant bactericidal effect under some specific conditions (Figure 6). When the phage concentration was 10^8^ PFU/m^3^, an exposure time of 10 min was not sufficient to inactivate *M. smegmatis* on an agar surface because more than 10% of the bacteria still survived at the three tested concentrations (Figure 6A). Extending the exposure time increased the bactericidal effect on *M. smegmatis* under all test conditions. An exposure time longer than 30 min may be needed to reach a satisfactory inactivation rate (>95%) and no bacterial colonies could be detected when they were exposed to the phage aerosols for 60 min (Figure 6A). In addition, increasing the airborne phage concentration reduced the survival rate of *M. smegmatis* (Figure 6B). All test groups revealed that 60 min of exposure time could make all colony counts of *M. smegmatis* nondetectable. Application of the highest concentration of phage aerosol (10^10^ PFU/m^3^) did not demonstrate a significantly higher bactericidal effect than those of lower concentrations (Figure 6C). A longer exposure time of 60 min was still needed to make the colony counts of *M. smegmatis* nondetectable. Overall, there was a significant difference in the bactericidal effect among the three exposure times (*p* < 0.001). Application of the phage aerosol for 10 min demonstrated a lower bactericidal effect than those after 30 min (*p* = 0.039) and 60 min (*p* < 0.001) of application, respectively. However, there was no significant difference in the bactericidal effect among the three different concentrations of ϕBTCU-1 aerosol or the three *M. smegmatis* concentrations.

## 4. Discussion

If ϕBTCU-1 aerosols are applied to the environment, one critical issue is their potential toxicity. The whole genome of ϕBTCU-1 has been fully sequenced [15] and no putative or confirmed toxin gene of ϕBTCU-1 phage has been found in the NCBI database. Furthermore, we also observed no prophage-related gene in ϕBTCU-1. Although it is reasonable to assume that no safety issues related to toxin production or chromosomal integration of ϕBTCU-1 may occur, nevertheless, mycobacteriophages display a great genetic diversity and nearly 50,000 genes can be sorted into 3900 groups and 75% of these genetic groups are of unknown function [22]. Therefore, to fully confirm the safety of ϕBTCU-1, a more complete database is still needed for comparison. Previous studies demonstrated that phage-encoded toxins may contribute to the virulence of several bacterial pathogens, including *Coynebacterium diphtheria*, *Escherichia coli*, *Salmonella* sp., and *Vibrio cholera* [22]. However, the pathogenesis of MTB is quite different from these bacteria and there is no evidence for mycobacteriophages-encoded toxins [22].

To maintain infectivity, it is recommended that viruses refrigerated at 4 °C not be used after 48 h [23]. This study showed that ϕBTCU-1 was stable for at least one week at 4 °C, but it would immediately lose infectivity after that time. The exact mechanism involved in the rapid decrease in phage viability is not known. However, the literature has also indicated that some phages are not stable at a refrigerated temperature, but are stable when stored at a temperature lower than 4 °C [24]. If phages are stored for longer periods, it is still recommended that they should be kept at −80 °C [25]. Cryoprotectants, such as glycerol and dimethyl sulfoxide (DMSO), may help viruses to maintain their infectious state [26]. However, it is not known whether these chemicals interfere with the bactericidal effect of phage aerosols. Therefore, the concentration of glycerol in our study (0.6%) was much lower than that applied in a previous study (5–10%) [26,27]. Our previous work demonstrated that the common procedure for phage aerosol decontamination is to conduct phage typing by using the patient’s bacterial specimen first and then choosing the most active phage(s) for amplification [14]. Based on our results in Figure 2, the phage solution used for aerosol generation would be used at an early date and would not be stored for more than a week.

For sampling airborne MTB or other surrogate species, the bioaerosol samplers used in this study had been previously applied. However, these samplers had never been compared to each other when they were chosen for sampling. A previous study suggested that decreased culturability determines the inactivation rate and that the type of sampler may influence the inactivation rate [28]. Therefore, it would be meaningful to understand which sampler is the most suitable for collecting culturable MTB in the air. This part of the study not only refers to obtaining the correct inactivation rates but also serves as a reference for collecting airborne culturable MTB in the indoor environment.

CR represents the CR of *M. smegmatis* aerosols collected on each bioaerosol sampler; samplers with higher CR values correspond to those that can preserve more culturable bacterial colonies. Based on the same CR indicator, the resistance of *M. smegmatis* to the stress from impaction and filtration was lower than that of endospores or *Penicillium* spores (CR = 0.15–0.38) [29], but similar to that of Gram-positive *S. aureus* (CR = 0.01–0.1) [30]. Apparently, resistance to different sampling stresses is species dependent. The CR values of *M. smegmatis* indicate that the BioSampler samples were superior to Nuclepore filtration, which was similar to the results of previous studies that sampled airborne *Candida famata, E. coli*, and *Legionella pneumophila* [31,32]. Even if the cell wall of the *Mycobacterium* species, which contains mycolic acid and is rich in lipids, is responsible for its resistance to desiccation, the stress from long-term air filtration can still reduce the culturability of *M. smegmatis*. In addition to Nuclepore filtration, a gelatin filter is also not suitable for collecting airborne *M. smegmatis* with a low concentration, because long-term sampling by filtration can result in more biological stress from dehydration during the sampling and extraction process [33]. This finding makes it not feasible to determine airborne MTB by culture assay when filtration sampling is conducted in the field.

Since MTB can suspend and spread through the air, we investigated the efficiency of ϕBTCU-1 aerosols in reducing airborne *M. smegmatis*. There have been very limited studies that have applied ultrasonic vibration to generate phage aerosols. A previous study employed a vibration frequency of 48 kHz to generate phage droplets and found a significant 2 log reduction in the phage concentration [34]. However, the phage concentration in our ultrasonic humidifier was stable for up to 60 min of aerosol generation. Our phage deposition test also agreed with this finding; ϕBTCU-1 survived well throughout the ultrasonic vibration process and was successfully deposited on the plate surface. This may be related to the fact that we kept the vibrating solution at a low temperature and applied a lower vibration frequency of 10 kHz to generate phage aerosols.

For airborne disinfection, we observed that a higher ϕBTCU-1 concentration of 10^9^ PFU/m^3^ is recommended to reduce *M. smegmatis* contamination. To our knowledge, no study has applied an airborne phage against airborne MTB or their surrogate species. For environmental control, UVGI is a well-known technology that effectively kills or inactivates airborne MTB. To achieve satisfactory efficiency, airborne MTB must be exposed to high UVGI doses. Riley et al. indicated that 90% of MTB and surrogate test organisms can be inactivated if these species are exposed to a UV intensity of 50 μW/cm^2^ for 12 s or 10 μW/cm^2^ for 60 s [35,36]. In comparison with UVGI, biocontrol-applied ϕBTCU-1 to inactivate *M. smegmatis* can also achieve the same efficiency within a few seconds. When *M. smegmatis* is suspended in the air, it can diffuse freely and may contact with ϕBTCU-1 from all directions. Consequently, if the generated phage concentration is high enough (at least higher than 10^8^ PFU/m^3^), the airborne phage can be easier to attach to their bacterial host and inactivate them in a short period of time.

For TB transmission, it is believed that the major route is through the inhalation of MTB aerosols, not by surface contact [37]. Although there is insufficient evidence to suggest that MTB can be reaerosolized from contaminated surfaces, MTB can still survive on surfaces for up to 4 months [38]. In addition, the MTB concentration on surfaces is likely to be related to the amount of airborne MTB, because bacteria may repeatedly aerosolize and settle during human activity [39]. Consequently, from an environmental control point of view, the question remains as to whether phage aerosols are capable of inactivating MTB on surfaces.

Based on the flow rate of entrance air toward the chamber, the chamber should be filled with phage aerosols after aerosol generation for nearly nine minutes. However, the phage deposition test demonstrated that 10 min of phage exposure time may still not be sufficient for all phage particles to cover the full surface area of the plates. The difference between the theoretical and actual times for phage particles to fully cover the plates may be related to the relatively large droplet size (5 μm) generated by the ultrasonic humidifier. Larger aerosol particles would take longer to distribute evenly in the chamber. Consequently, to achieve a satisfactory inactivation efficiency greater than 90%, agar surfaces contaminated with *M. smegmatis* were often exposed to the phage for longer than 10 min, depending on the phage and *M. smegmatis* concentrations.

For surface inactivation, we observed that the ϕBTCU-1 concentration required to reduce *M. smegmatis* contamination was higher on the agar surface than in the air. As *M. smegmatis* does not diffuse as freely on an agar surface as in the air, a higher concentration of ϕBTCU-1 and a longer exposure time were required for the phages to contact their host and attach well. Moreover, the bacteria spread on the agar surface may aggregate, although we spread *M. smegmatis* on the agar as evenly as possible. These possible reasons may explain why higher phage concentrations and longer exposure times were needed for the inactivation of *M. smegmatis* on the surface than in the air. In comparison with UVGI, Collins found that MTB on an agar surface exposed to 40 μW/cm^2^ UVC for 120 s would result in a 2 log reduction [40]. Under the same colony reduction efficiency, *M. smegmatis* on an agar surface had to be exposed to ϕBTCU-1 aerosol for at least 30 min in our study. UVGI is more efficient for surface disinfection than applying phage aerosols. However, microbe growth could occur in crevices and UVGI may not penetrate these shadowed areas. Moreover, UVGI may damage surface materials and it can also cause diseases such as skin erythema and photokeratitis [41]. Applying phage aerosols for disinfection may overcome these limitations; aerosols can reach the crevices and no evidence suggests that phages will negatively impact human health. Consequently, applying phages as environmental biocontrol agents may be an alternative approach if other decontamination methods are difficult to implement.

The major purpose of this study was to determine the phage concentration and aerosol generation time during aerosol decontamination. To simplify our experimental conditions, a single phage, ϕBTCU-1, was applied. Most lytic phages can kill their bacterial host via synthesis and assembly of new phage and then lysis of its host. Although it’s difficult to determine whether ϕBTCU-1 can only kill their host by lysis, but without the multiplication process, our previous study demonstrated that the endolysins derived from ϕBTCU-1 have antimycobacterial activity [15]. Thus, ϕBTCU-1 may be a good candidate for disinfectant agents. Nevertheless, it may be impractical to conduct aerosol decontamination by a single phage. From our previous experience with CRAB decontamination by phage aerosols, we conducted phage typing and selected the optimal phage for aerosol decontamination [14]. Phase typing was carried out after we obtained the patient’s clinical specimen. For aerosol decontamination, only the most active single phage or phage cocktail is selected, based on the score of the lysis zone of the patient’s bacterial samples by using the agar overlay method [14]. Since only a few phages can effectively kill MTB, previous studies have recommended a combination of three to six phages to reduce the possible emergence of phage-resistant bacteria [22]. Therefore, in the future, we will continue to isolate more phages such as ϕBTCU-1 when we apply phages for environmental decontamination in the ward.

When applying phage aerosols for the inactivation of bacteria in the environment, another concern is whether the physiological status of bacteria can affect phage infection efficiency. Bacteria displaying various physiological statuses can engage in phage and host interactions and may increase the risk of phage resistance [42]. Similar to most in vitro studies, our study also performed an experiment with a stationary phase culture of *M. smegmatis*. However, the knowledge of MTB physiology in the environment is very limited due to its slow growth. Consequently, the use of phage aerosols still needs to coordinate with other environmental cleaning methods or the use of phage cocktails to decrease the potential risks of phage resistance.

To date, thousands of phages have been isolated using *M. smegmatis* mc^2^155 [22]. However, only one or two clusters of mycobacteriophages can efficiently infect MTB and relatively few mycobacteriophages can kill MTB [22]. Very few mycobacteriophages, including ϕBTCU-1, have been fully investigated to demonstrate their nontoxicity in Taiwan, so it is difficult for us to conduct aerosol disinfection by phage cocktails at present. However, our study still demonstrated the feasibility of bacteriophage-containing aerosols for disinfection and established an evaluation method for determining the biocontrol efficiency. Although phage therapy of TB is still difficult, mycobacteriophages are an alternative to prevent disease transmission, rather than to treat disease [43]. The application of phage aerosols may be a cheap and safe way to disrupt disease transmission.

## 5. Conclusions

Antibiotic-resistant MTB has become an important issue in public health. Many control techniques have recently been developed for controlling MTB in the air. However, some of these techniques could be harmful to human health. As MTB are resistant to currently available antibiotics or sanitizers, phages may represent an alternative decontamination approach. Our results suggest that the phage aerosol may be used as an environmental biocontrol agent to decontaminate MTB in the air or on the surface. However, a high phage concentration (>10^9^ PFU/m^3^) is required to ensure that airborne phages can efficiently contact their hosts. Under a high phage aerosol concentration, an aerosol generation time of 60 min is recommended to achieve an optimal bactericidal effect on MTB.

## Supplementary Materials

The following are available online at https://www.mdpi.com/2076-2607/7/8/237/s1, Figure S1. Stability of culturable *M. smegmatis* in the Collison three-jet nebulizer. Figure S2. Stability of the culturable phage ϕBTCU-1 in the ultrasonic humidifier. Figure S3. Stability of culturable ϕBTCU-1 phage in the air of test chamber.
