# Supplementary figures and images for: Development of a Biocontrol Method Applying Bacteriophage-Containing Aerosol against Mycobacterium tuberculosis Using the Bacteriophage BTCU-1 and M. smegmatis as Models
Source: Microorganisms. 2019 Aug 3;7(8):237. doi: 10.3390/microorganisms7080237 (PMC6722745; doi:10.3390/microorganisms7080237)

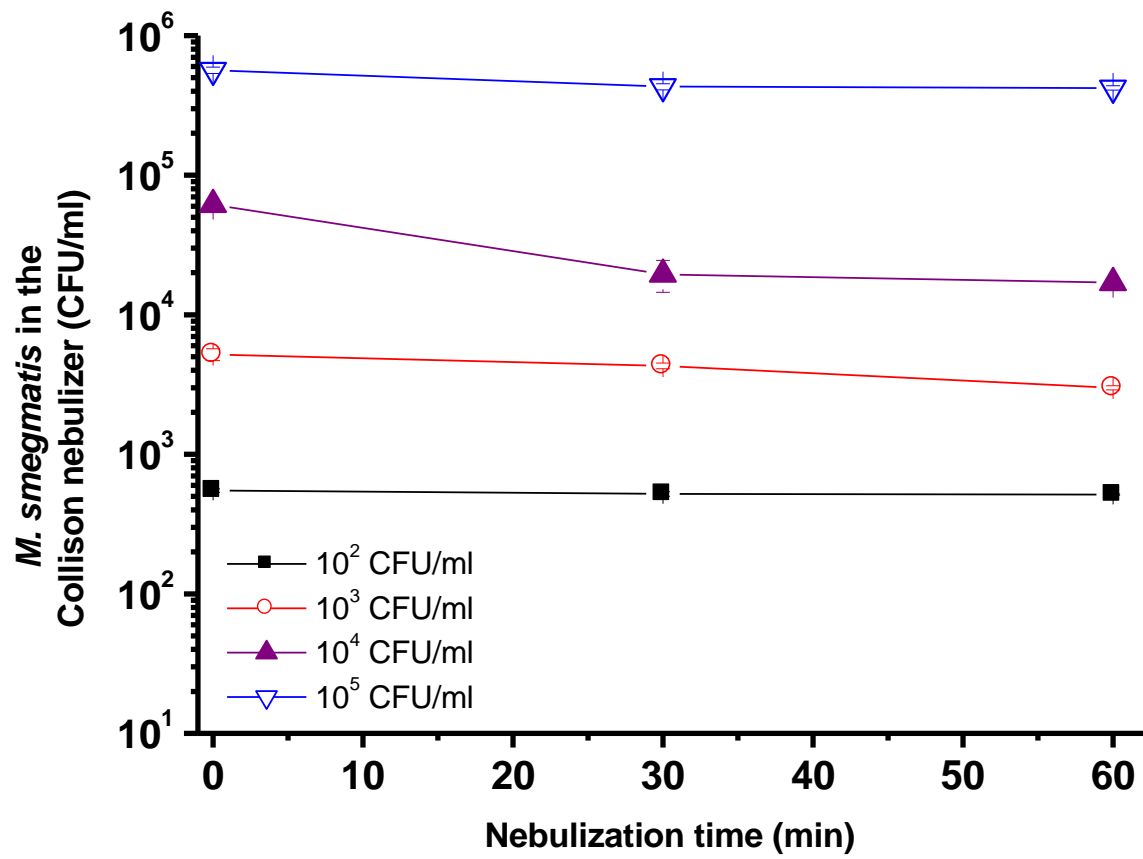

**Figure S1.** The stability of culturable *M. smegmatis* in the Collison three-jet nebulizer.

Supplement: Supplementary file 1 [file microorganisms-07-00237-s001.zip › C5FMVNQT_Supplementary_Material_Figure_1_ed_final je.pdf]

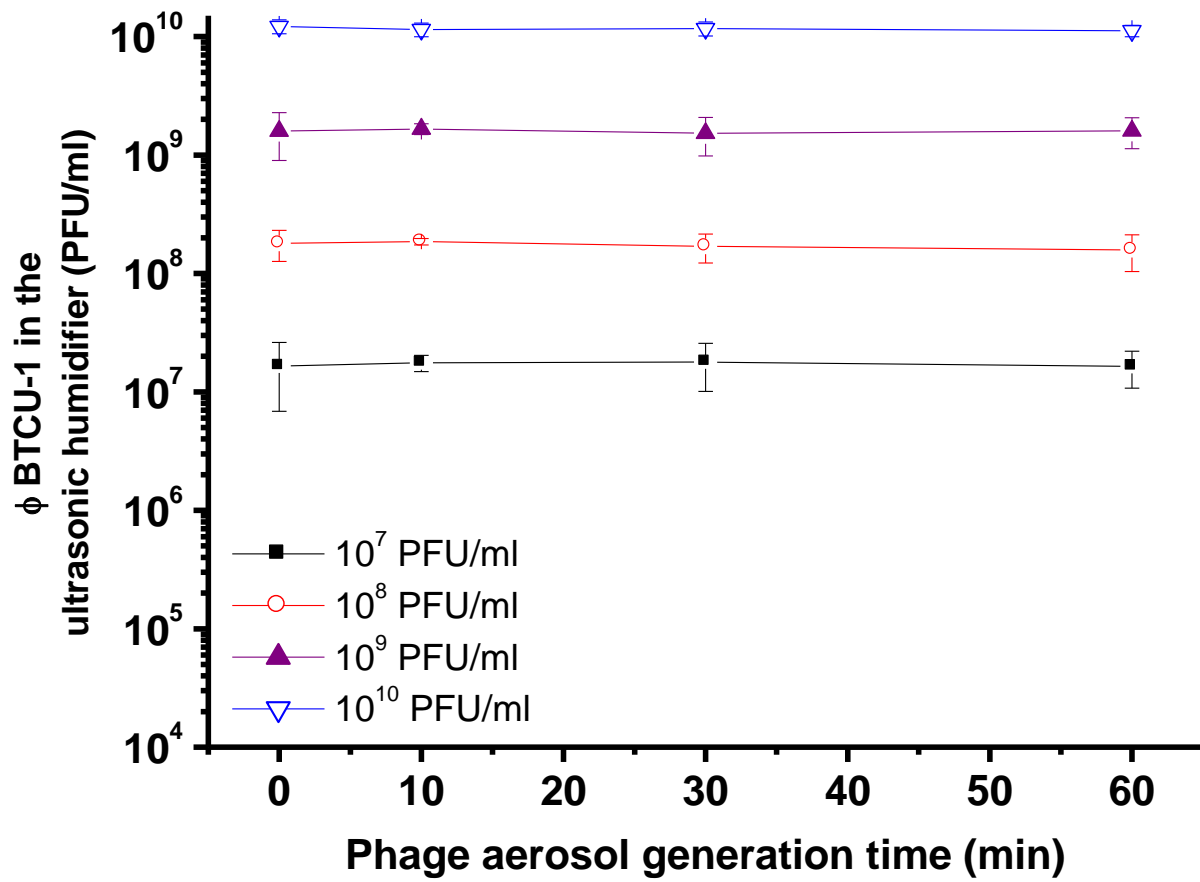

**Figure S2.** The stability of culturable phage  $\phi$ BTCU-1 in the ultrasonic humidifier.

Supplement: Supplementary file 1 [file microorganisms-07-00237-s001.zip › C5FMVNQT_Supplementary_Material_Figure_2_ed_final je.pdf]

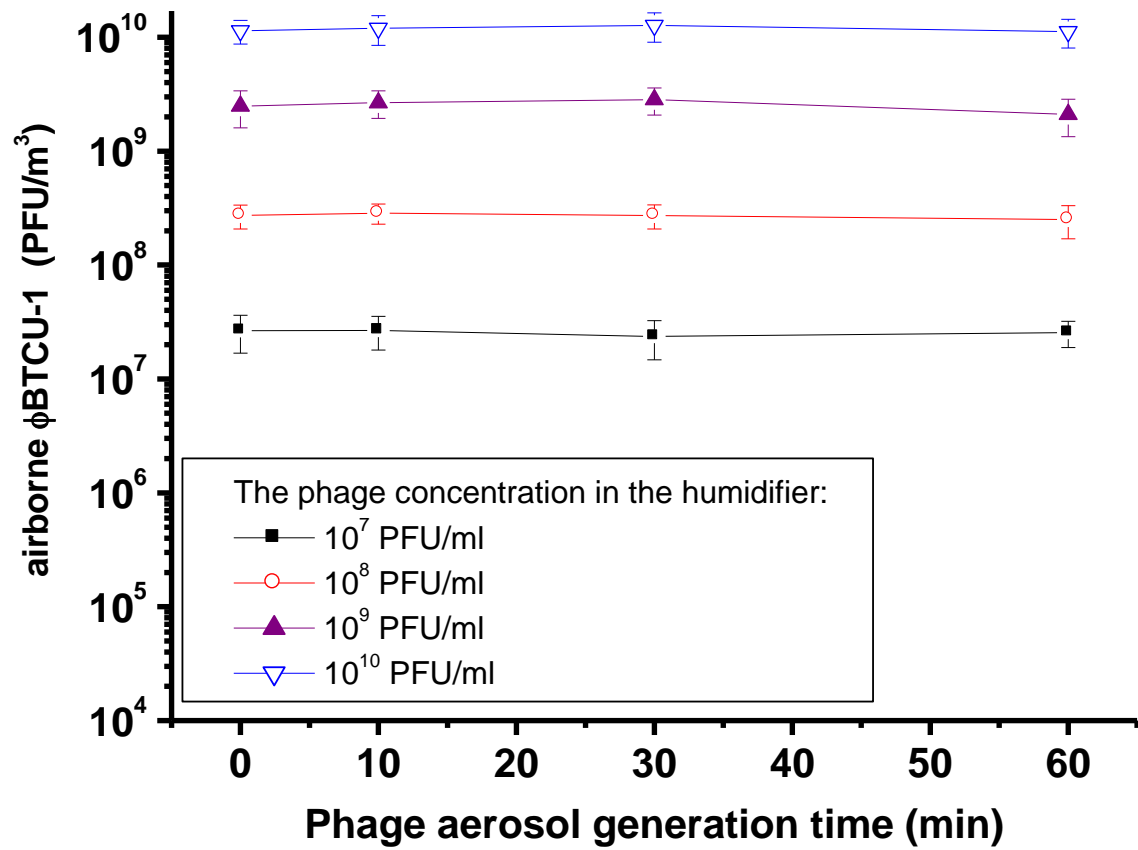

**Figure S3.** The stability of culturable phage  $\phi$ BTCU-1 in the air of test chamber.

Supplement: Supplementary file 1 [file microorganisms-07-00237-s001.zip › C5FMVNQT_Supplementary_Material_Figure_3_ed_final je.pdf]
